# Distribution of Rare Earth Elements in Ash from Lignite Combustion in Polish Power Plants

**DOI:** 10.3390/ma17184477

**Published:** 2024-09-12

**Authors:** Zdzisław Adamczyk, Joanna Komorek, Magdalena Kokowska-Pawłowska, Jacek Nowak

**Affiliations:** Faculty of Mining, Safety Engineering and Industrial Automation, Silesian University of Technology, Akademicka 2A, 44-100 Gliwice, Poland; joanna.komorek@polsl.pl (J.K.); magdalena.kokowska-pawlowska@polsl.pl (M.K.-P.); jacek.nowak@polsl.pl (J.N.)

**Keywords:** lignite, fly ash, bottom ash, rare earth elements, yttrium, REY, critical raw materials, amorphous glass

## Abstract

Rare earth elements are an essential critical raw material in the development of modern technologies and are highly sensitive to both supply chain disruptions and market turbulence. The presented study examines the characteristics of fuel, fly ash, and bottom ash from lignite combustion in power plant units. Also, we attempted to determine the amount of amorphous glass in the ashes and whether and to what extent the glass from the ash samples is bound to REY. The suitability of the ash was assessed as an alternative source of REY. The fuel and ash samples were acquired from power plants in Poland. The tests determined the fuel quality parameters, including the chemical and phase composition, of amorphous glass using ICP-MS and XRD methods, respectively. The study showed that all ash samples dissolved in 4% HF were enriched in REY. The efficiency of REY enrichment varied, and its presence in the residue samples was found to be in similar proportions compared to the raw sample. All ash residue samples were enriched in critical elements. The obtained values of the C_outl_ prospective coefficient allowed for the classification of some of the analyzed ashes and their residues after dissolution in 4% HF as prospective REY raw materials.

## 1. Introduction

The development of modern technology is based on the use of many mineral-based raw materials. Rare earth elements (REEs) are a special group, which includes 17 elements, 15 of which belong to the lanthanide group, and the remainder are scandium and yttrium. The addition of yttrium to the rare earths elements (REY) allows them to be divided into the following three subgroups: light (LREY: La, Ce, Pr, Nd, Pm, and Sm), medium (MREY: Eu, Gd, Tb, Dy, and Y), and heavy (HREY: Ho, Er, Tm, Yb, and Lu). The above classification is very convenient for describing the distribution of rare earth elements and yttrium (REY) in coal as well as in coal ash and ores [1,2,3].

REY are an essential raw material in the development of modern technologies. They have been applied in electronics, electrical engineering, electromobility, green energy extraction, and many other industries [4,5]. These metals are classified as critical raw materials because they lack primary and secondary sources without which the development of modern technologies is impossible. These raw materials are characterized by high supply risk and great economic importance. In European Union countries, REEs are a highly sensitive raw material to the supply chain and to market turbulences [6,7,8,9,10,11]. All available forecasts point to a rapidly growing demand for REY and other critical raw materials [12,13,14]. The Organization for Economic Co-operation and Development projections show that demand for critical raw materials will increase from 79 billion tonnes in 2023 to 167 billion tonnes in 2060, and for REY alone, it will be 7 times higher by the end of the next decade [15,16,17].

As the demand for REY increases, alternative sources of these elements have been explored, such as apatite phosphogypsum, electronic wastes, and ash from coal and lignite combustion. In addition, reports have examined the development of new methods and technologies for the recovery of REEs [3,18,19,20,21,22,23,24,25,26,27]. The REY content in fly ash (FA) and bottom ash (BA) generated from the combustion of hard coal and lignite may range from several dozen to several hundred ppm, and rarely reaches approx. 1% [28,29,30,31,32,33,34,35,36]. However, studies have scarcely simultaneously tested FA and BA from the same fuel batch’s combustion cycle.

The Polish energy sector is based on the processing of fossil fuels, especially hard coal and lignite. The production of energy from fossil fuels generates large amounts of waste, which should be recycled. These include FA, slag, ash–slag mixtures, microspheres, and waste from flue gas desulfurization [27,37,38,39,40].

The combustion of lignite and hard coal in power boilers is associated with an increase in the content of trace elements, including REY, in the power wastes compared to the fuel. Coal ash can serve as a secondary raw material in various industries (building materials, ceramics, mining, etc.) and is also considered a promising source of REY, which is of great strategic importance to Poland, as it does not have deposits of these elements of economic importance [3,21,32,41,42,43,44,45,46,47].

In coal, REEs are most commonly found in association with minerals. Potential carriers of REEs in coal may be phosphates, clay minerals, and some sulfides. However, other studies have shown that REEs in coal can be associated with both organic and inorganic matter. In the case of FA’s composition, minerals from the aluminosilicate group are most common. The composition of BA is similar to that of FA, but it contains minerals enriched in high-density elements such as Fe and Mn. Both FA and BA are enriched in REY. Mineralogical analyses of Polish and worldwide coal ash show that FA can contain up to >70% amorphous glass and <30% mineral phases such as mullite, quartz, and iron oxides [27,28,32,48,49].

Coal will continue to be the main source of energy in the world in the coming years. For this reason, the amount of waste from the power industry will increase. The global level of disposal of this waste is not satisfactory, although, in some countries, the utilization rate is 100% (Denmark, Italy, and The Netherlands) [49]. The complex composition, fine size, and variable particle morphology and properties of ash complicate its optimal use. Furthermore, ash of the same type, but derived from coal from different coalfields, has different suitability for REY extraction processes [50]. One possible utilization of energy waste may be the obtainment of REY.

The aim of our study was to characterize the distribution of REY in the fuel, FA, and BA from lignite combustion in the power units of two Polish power plants, Bełchatów and Turów. The distribution of REY was determined based on the concentration of these metals in FA from the electrofilter, from the flue gas duct, and in BA, which originated from the same fuel combustion cycle. The study also attempted to determine the amount of amorphous glass in the ashes and whether and to what extent the glass from the ash samples is bound to REY.

## 2. Selection of Samples and Research Methodology

The study examined four fuel samples—lignite and 11 samples of ashes from the combustion of this fuel in the Bełchatów and Turów power plants in Poland (Table 1). FA samples were acquired from the electrofilter (Bełchatów and Turów) and the gas duct (Bełchatów) and together with BA samples (Bełchatów and Turów) originating from the same combustion cycle. The test results of the samples of energy waste obtained from the same combustion cycle of a portion of fuel allow for the demonstration of the variability of REY concentration for the potential of their recovery.

The coal samples were reduced and grinded to the φ < 1 mm fraction and briquettes were made for microscopic examination of the petrographic composition of coal samples. Petrographic analysis was carried out according to the International Committee for Coal Organic Petrology (ICCP) recommendations taking into account Polish standard PN-ISO-7404 [51]. For microscopic studies, a Zeiss reflected light microscope was used. The remaining samples were grinded to the φ < 0.2 mm fraction for proximate analysis. For all coal samples, the content of ash (A^a^), moisture (W^a^), and calorific value (Q_s_^af^) were determined according to Polish standards PN-ISO 1171:2002 [52], PN-ISO 687:2005 [53], and PN-ISO 1928:2020-05 [54], and petrographic analyses were performed.

Coal ash contains crystalline mineral components and amorphous glass, which differ in chemical reactivity. Hence, reports have examined the dissolution of amorphous glass from coal ash using hydrofluoric acid (HF) [32]. In this case, 10 g of each ash sample was mixed with 200 cm^3^ of 4% HF solution in a 250 cm^3^ high-density polyethylene (HDPE) bottle. The mixture was shaken and then left for 24 h at room temperature (22–25 °C). The solid residue was repeatedly washed with deionized water (until a neutral reaction mixture was obtained). The samples were then dried in a thermostatic oven at 60 °C until a constant mass was obtained.

The ashes and their residue after dissolution in 4% HF provided test material for the determination of chemical composition, including REY and phase composition of, for example, amorphous glass. The chemical composition was determined by ICP-MS (inductively coupled plasma mass spectrometry) using a Perkin Elmer SCIEX ELAN 6000 ICP-MS spectrometer at Activation Laboratories Ltd. in Ancaster, ON, Canada. Loss on ignition (LOI) was determined by weight in accordance with ASTM D7348-21 [55]. The phase identification measurements were performed on a Bruker Phaser 2 diffractometer (XRD) under the following conditions: Cu_Kα_ radiation, 2θ angle range from 5° to 75°, step of 0.01°, time of 1 s, and internal standard of ZnO, which was added to the sample at a rate of 12%. Identifying the content of the mineral phases and amorphous glass was possible using the Rietveld method with Topas V7.0 software. To characterize the mineral composition of tested ash samples, including the proportion of amorphous glass, the unburnt carbon content (UC) must be known. The simplest method to obtain the UC in the studied samples was by determining the LOI content of the ash.

## 3. Results

### 3.1. Fuel Properties

The Bełchatów power plant burns low-rank B fuel—metalignite. According to the United Nations’ Economic Commission for Europe (UN-ECE) Geneva 1998 classification of in-seam coals [56], tested samples represented medium-grade coal (samples BW1 and BW2) or low-grade coal (sample BW3). The petrographic composition of the analyzed samples was dominated by macerals of the huminite group (Table 2). Among them, humocolinite, corpohuminite, atrinite, and densinite were observed. The content of macerals from the liptinite group did not exceed 5% and was represented by resinite or sporinite. The content of the inertinite group varied from 4% to 6% and was mainly represented by inertodetrinite and less frequently by fusinite. The samples were characterized by a high mineral matter content ranging from 19% to 23%. The mineral matter was mainly represented by fine-grained polymineral substances. Iron sulfides, often in the form of framboidal pyrite, and carbonates were also observed in the samples.

The Turów power plant burned low-rank A fuel—subbituminous coal. According to the UN-ECE Geneva 1998 [56], the sample represented low-grade coal. The petrographic composition of the TW4 sample was dominated by macerals of the huminite group (Table 2), which were represented by humocolinite, textinite, corpohuminite, artinite, and densinite. The maceral content of the liptinite group was 6%. Liptinite was represented by sporinite and resinite. The content of the inertinite group was 5% and was mainly represented by inertodetrinite, semifusinite, and funginite. The mineral matter was mainly represented by fine-grained polymineral substances, iron sulfides, and carbonates.

This type of coal is commonly used as fuel in large power plants [47].

### 3.2. Chemical Composition

The chemical composition of the ash indicates that despite the combustion of lignite of different quality in both power plants (Bełchatów and Turów), and regardless of the sampling location (FA from the electrofilter or gas duct, and BA), their dominant chemical components were SiO_2_, Al_2_O_3_, and CaO (Table 3). These components were present in amounts of several dozen or a dozen percent, constituting a total content in the range of 75–85%, with the lowest being for BA and the highest being for FA from the electrofilter, while FA from the gas duct had intermediate values. Fe_2_O_3_ was also an important quantitative component, and its contents in BA and FA from the gas duct were similar (4.15–6.71%), while FA from the electrofilter was higher (5.55–7.46%). The contents of the remaining chemical components occurred in much smaller amounts. An important component of the tested ashes was LOI, and its highest amounts were found in BA and FA from the gas duct from B, and the lowest in FA from the electrofilter in both power plants. According to the literature, the chemical compositions presented are typical for this type of fuel [10,45,48,49].

**Figure 1:**
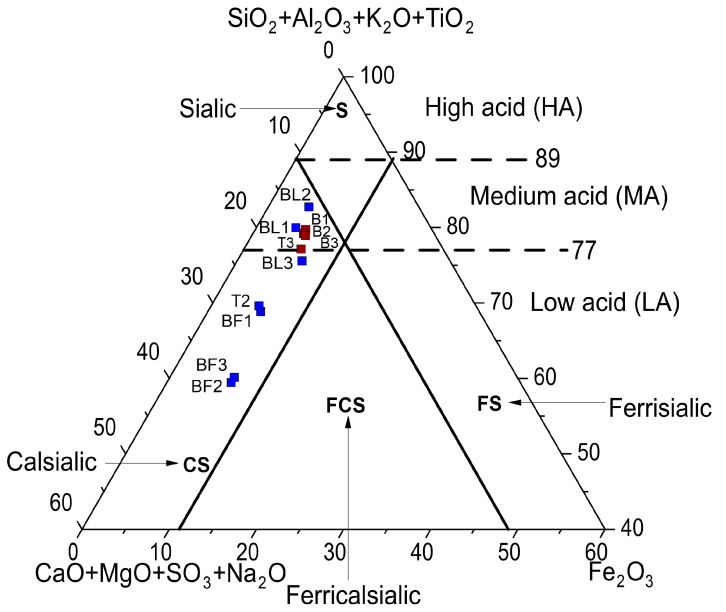
Analyzed ash samples compared to Vassilev’s chemical classification of the inorganic matter in coal ash [58]. Explanations: blue symbol—fly ash, brown symbol—bottom ash.

The chemical diversity of the tested ashes is best shown in the chemical classification of inorganic matter in coal ashes introduced by Vasiliev [57], based on the standardized content of the main oxides. In this classification, all BAs were of the calsialic–medium-acid (CS-MA) type (Figure 1), while FAs from the flue gas duct were of the calcareous–medium-acid (CS-MA) type—samples BL1 and BL2—or calcareous–low-acid (CS-LA) type—sample BL3. FA samples from the electrofilter were classified as calsialic–low-acid (CS-LA) type.

The SiO_2_/Al_2_O_3_ ratio values were the highest in BA and FA from the gas duct from Bełchatów (2.37–3.37), and the lowest in FA from Bełchatów and BA and FA from Turów (1.42–2.12) (Table 3). Also, the K_2_O/Na_2_O ratio values varied, where the highest value was in BA from Turów (2.93) and the lowest was in FA from the electrofilter. The (MgO + CaO)/(K_2_O + Na_2_O) ratio was in a wide range (7.20–125), with the highest values occurring in FA from the electrofilter from Bełchatów (above 82), and the lowest in BA and FA from Turów (below 8). In the case of the CaO/MgO ratio, the values were in the range of 11.68–25.19. The highest values of this ratio were observed in FA from the Bełchatów electrofilter, and the lowest in FA from Turów. BA and FA samples from the gas duct showed similarly high values of the detrital/autogenous index (DAI) (3.06–4.63), and the lowest ratio was characteristic of FA from the electrofilter (1.45–2.42). DAI values indicate enrichment of ash with detrital components [57].

### 3.3. Mineral Composition of Ash Samples

XRD results showed differences between the phase composition of the BA and FA from the electrofilter and gas duct (Table 4). In all ashes, amorphous glass with quartz dominated. The highest amounts of glass were observed in BA and FA from Turów (66.3–70.7%) and FA from the electrofilter from Bełchatów (51.7–59.5%). This was accompanied by the lowest amounts of quartz. The lowest amounts of glass were in BA and FA from the gas duct from Bełchatów (30.1–47.5%), with the highest amounts of quartz. The remaining phase components, i.e., mullite, gehlenite, calcite, anhydrite, anorthite, maghemite, hematite, and lime, were not always present in the tested samples. In some cases, we observed significant amounts (e.g., mullite in FA from gas duct—12–20%), and in others (e.g., hematite and lime), they were present only in FA from Turów. The literature shows that the phase composition of the tested ashes is typical for the combusted lignite [10,43,44,48].

**Figure 2:**
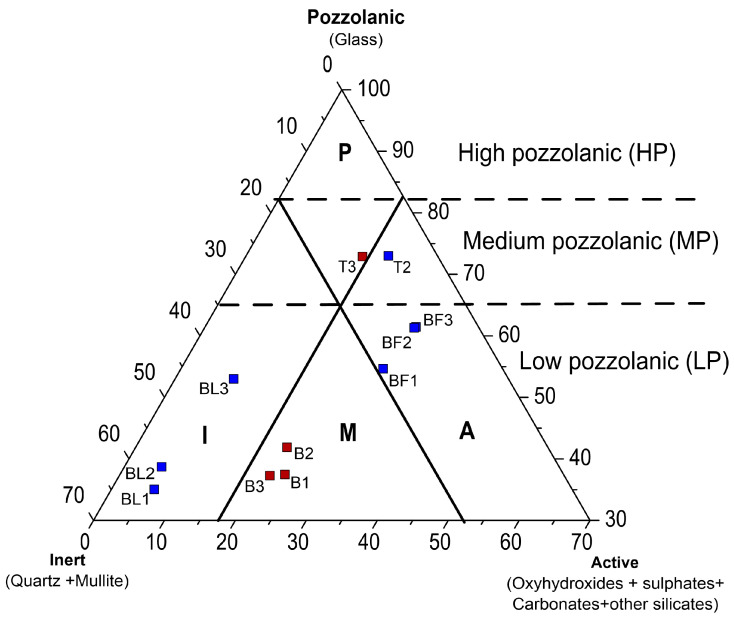
Vassilev’s phase mineral classification system [57]. Explanations: blue symbol—fly ash, brown symbol—bottom ash.

The phase composition of the tested ashes was compared with the Vassilev phase mineral classification system [58]. On this basis, BA from Bełchatów was classified as mixed–low pozzolanic (M-LP), and the BA from Turów as pozzolanic–medium pozzolanic (P-MP). FA from the gas duct was also classified as inert–low pozzolanic (I-LP), FA from the electrofilter as active–low pozzolanic (A-LP), and FA from Turów as active–medium pozzolanic (A-MP) (Figure 2).

### 3.4. Rare Earth Elements and Yttrium (REY) in Ash Samples

**Figure 3:**
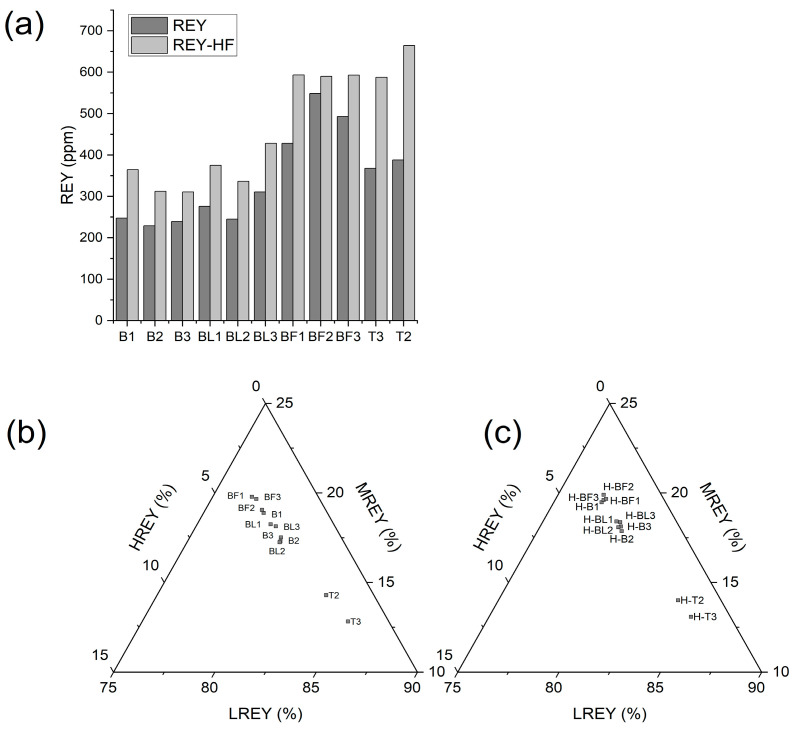
REY distribution in the tested samples: (**a**) the REY content of raw ash and residue samples dissolved in 4% HF; content of HREY and MREY; (**b**) percentage share of LREY, MREY, and HREY in raw ash; (**c**) percentage share of LREY, MREY, and HREY in residue samples after dissolution in 4% HF.

**Figure 4:**
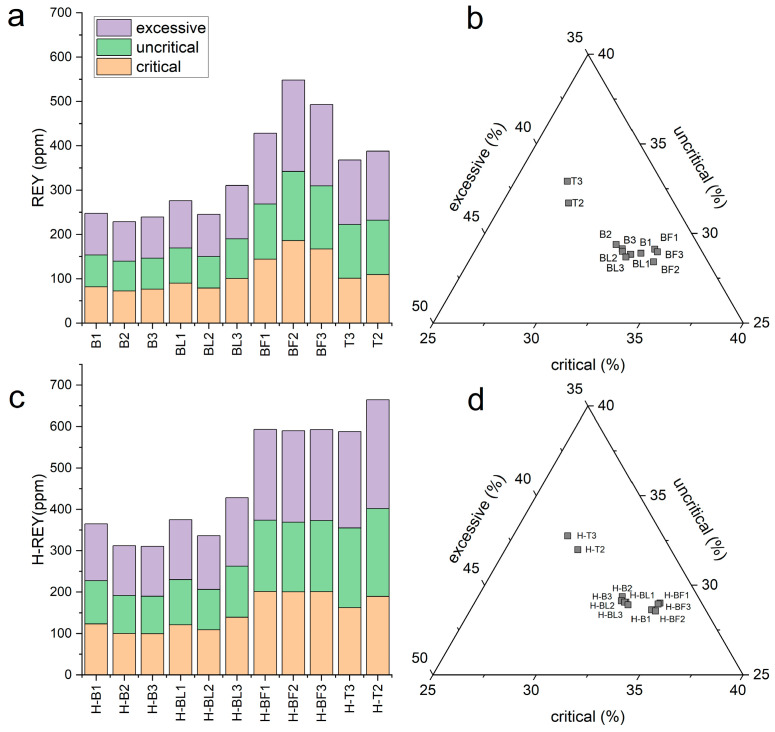
REY distribution in the tested samples: (**a**) content of critical, uncritical, and excessive elements in raw ash; (**b**) percentage share of uncritical and excessive elements in raw ash; (**c**) content of critical, uncritical, and excessive elements in residue samples after dissolution in 4% HF; (**d**) percentage share of uncritical and excessive elements in residue samples after dissolution in 4% HF.

The highest REY contents were found in FA from electrofilters (388.0–548.5 ppm) and the lowest in BA from Bełchatów (228.9–247.6 ppm). Among REY, LREY dominated and constituted 77.0–85.2%, while HREY had the smallest share in the range of 2.0–3.3% (Table 5, Figure 3a,b). FA samples from the electrofilter from Bełchatów (BF1–BF3) had higher amounts than the average for global deposits (404 ppm), while the remaining samples showed lower concentrations, but they were typical for ash [3,9,10]. Critical (Nd, Eu, Tb, Dy, Y, and Er) and uncritical (La, Pr, Sm, and Gd) elements were present in the ash in similar ranges, 72.5–186.2 ppm (27.5–33.9%) and 67.3–155.9 ppm (28.4–32.9%), respectively, while slightly higher amounts were represented by excessive (Ce, Ho, Tm, Yb, and Lu) elements—94.1–206.4 ppm (37.2–40.1%) (Figure 4a–d). The highest concentrations of these elements were found in FA from the electrofilter from Bełchatów and BA from Turów. The share of critical elements was similar to that of other lignite ashes from Polish power plants [10].

In order to assess the ash as an alternative source of REY, the prospective coefficient (C_outl_) was calculated, taking into account the shares of critical and excessive elements, according to the following formula [2,3]:

$$C_{outl}=\frac{Nd+Eu+Tb+Dy+Er+Y}{Ce+Ho+Tm+Yb+Lu}$$

**Figure 5:**
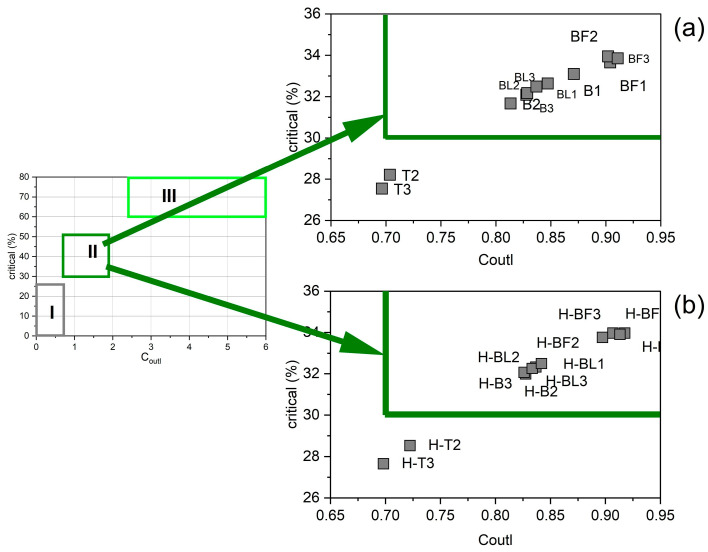
The relationship between the percentage share of critical elements and the C_outl_ prospective coefficient compared to the classification of coal ashes enriched in REE [3] (**a**) in raw ash samples and (**b**) in residue samples after dissolution in 4% HF. Source of REY: I—not prospective; II—prospective; III—highly prospective source of REY.

Additionally, to determine the potential industrial value, a graph was prepared describing the relationship between the percentage share of critical elements to C_outl_. The values of the C_outl_ for the tested ashes indicate that BA, FA from the gas duct, and FA from the electrofilter from Bełchatów are REY prospective raw materials, while BA and FA from Turów were not REY prospective raw materials (Table 5; Figure 5a).

The degree of enrichment of samples in REY in relation to their content in the upper continental crust (UCC) was determined by ensuring that the REY shares marked in the samples were normalized to their shares in the UCC. Regarding the distribution of REY content compared to the UCC, the samples were divided into enriched in LREY (L-type), enriched in MREY (M-type), and enriched in HREY (H-type). The normalization diagram of each type had a positive or negative anomaly with different amplitudes in Ce, Eu, and Y, because the environmental behavior of these elements may be different from other REY. Samples with an L-type REY content distribution were distinguished when the La_N_/Lu_N_ ratio was >1. Samples with an M-type REY content distribution were identified when the La_N_/_SmN_ ratio was <1, and the Gd_N_/Lu_N_ ratio was >1. The samples with an H-type REY content distribution were distinguished when the La_N_/Lu_N_ ratio < 1. Subtypes and intermediate types were distinguished by the presence of anomalies [3,22].

**Figure 6:**
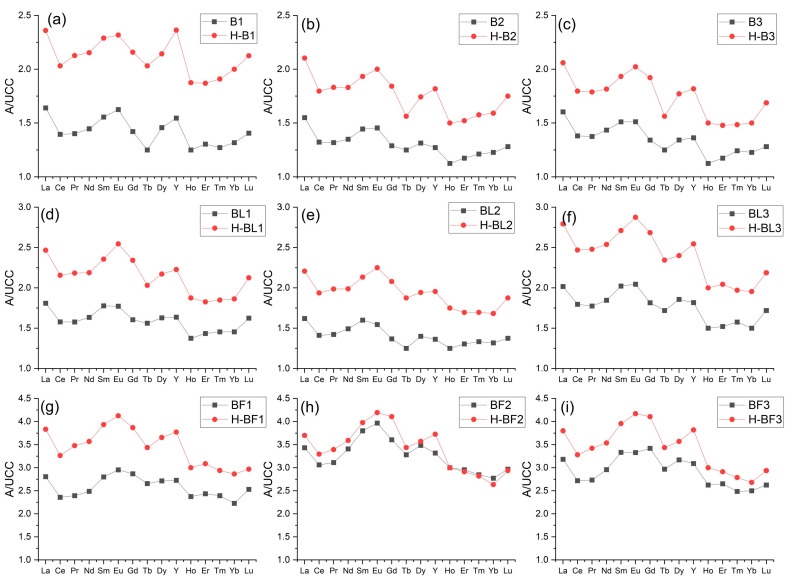
Distribution of REY content in the tested ash samples from Bełchatów power plant. The share of REY was normalized to their content in the UCC [59]. Explanation: black curve—raw sample, red curve—residue sample. (**a**–**i**)—sample symbols in the upper right corner.

**Figure 7:**
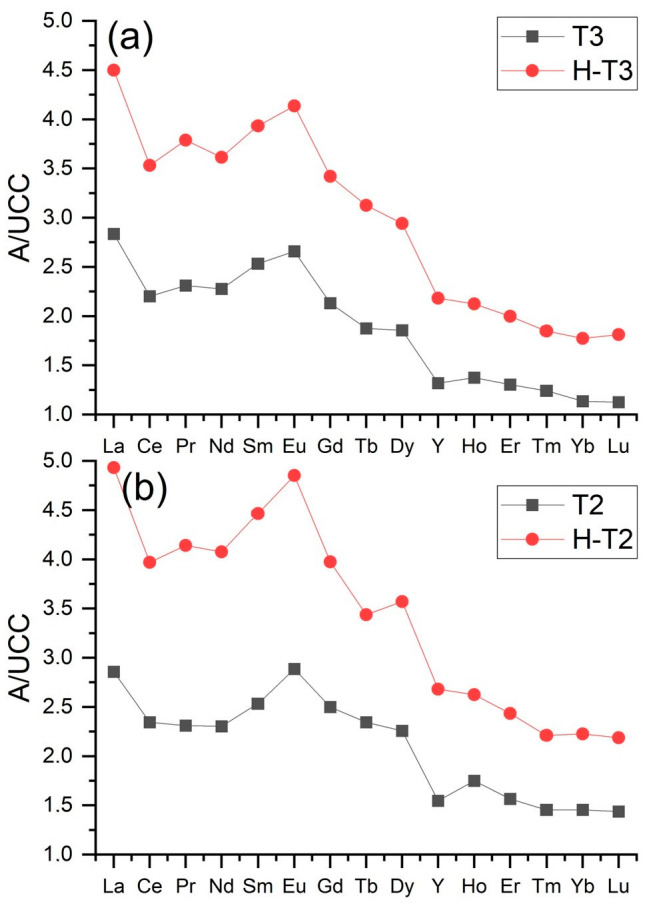
Distribution of REY content in the tested ash samples from the Turów power plant. The share of REY was normalized to their content in the UCC [59]. Explanation: black curve—raw sample, red curve—residue sample. (**a**,**b**)—sample symbols in the upper right corner.

The normalization curves for the lignite combustion ashes had a similar shape and were L- and L-M-type curves. These curves were above the reference level throughout their range. The content of some REY in the samples was up to three times higher than in the UCC (Figure 6 and Figure 7). All normalization curves of ash from Bełchatów showed two positive anomalies, larger for Eu and smaller for Y. The normalization curves of ash from Turów showed a significant positive anomaly for Eu and a negative smaller anomaly for Y. The positive Eu anomalies are attributed to the higher sorption of MREY in organic matter compared to LREY and HREY [3,24].

It should be noted that the total mass of lignite combustion ash in Bełchatów consisted of 10% BA (samples B1–B3), 1% FA from the flue gas duct (samples BL1–BL3), and 89% FA from electrofilters (samples BF1–BF3). Therefore, FA from electrofilters may represent a potential source of REY due to its largest share in the total mass of ash produced in B.

### 3.5. Characterization of the Residue after Ash Dissolution in 4% HF

The chemical composition of the raw ash was compared with the ash treated with 4% HF. The dominant chemical constituents in these residues after dissolving the tested ashes in 4% HF were SiO_2_, Al_2_O_3_, and CaO. In the ashes from B, their total content was over 60% and from Turów over 73%. Therefore, the SiO_2_ removal efficiency in BA varied and amounted to 20.46–22.74% in the samples from Bełchatów and 17.46% in the sample from Turów (Table 6). The highest SiO_2_ removal efficiency was demonstrated by FA from the electrofilters from Bełchatów and amounted to 48.78–59.90%, while FA from Turów was 35.83%, and FA from the gas duct ranged from 19.57 to 30.43%.

**Figure 8:**
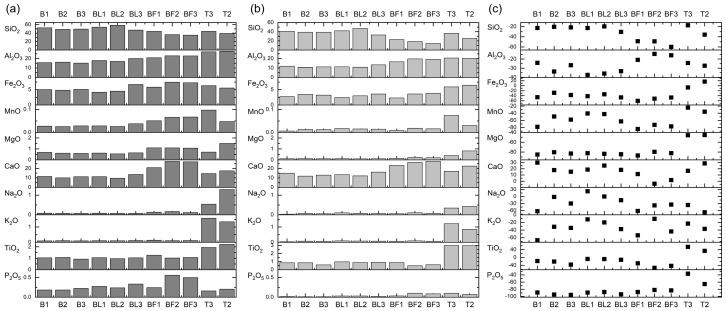
Comparison of the contents (%) of the main chemical components in (**a**) the raw ashes and in (**b**) the ash after 4% HF treatment; (**c**) enrichment or removal efficiency in (%).

Fe_2_O_3_ contents decreased in all ashes from Bełchatów in the residue after dissolution in 4% HF and also in BA from Turów (removal efficiency was 6.66–60.08%) Only for FA from Turów did the Fe_2_O_3_ content increase. In almost all samples from Bełchatów and FA from Turów, the CaO content increased (enrichment efficiency from 1.90 to 29.90%) (Table 6; Figure 8).

**Figure 9:**
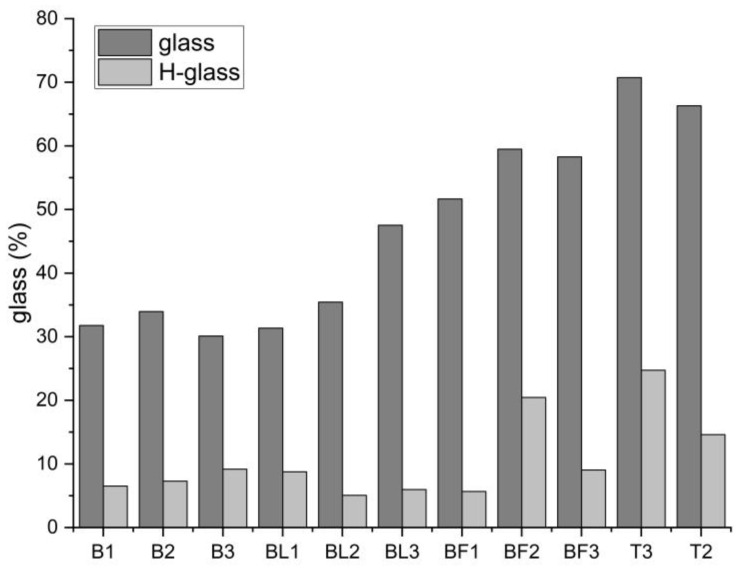
Distribution of amorphous glass content in raw ash and residues dissolved in 4% HF.

The results showed that the glass content was lower than in the raw samples and usually did not exceed 10% in ashes from B. In the case of ashes from Turów, their content ranged from 14.61 to 24.73%, but the amount of glass in these samples was the highest of all samples analyzed (>65%). In summary, the glass removal efficiencies in the tested ashes ranged from 65.04% to 89.01% (Table 6, Figure 9).

Quartz and calcite were present in all ash residue samples after dissolution in 4% HF and their content was higher than in the raw samples. This was probably due to the concentration of HF being too low to dissolve more silicate phases, including quartz. Mullite and anorthite were also found in most of the FA and FA from B.

The study showed that all residue samples were enriched in REY and ranged from 310.6 to 664.6 ppm. The highest REY contents were found in the ash from Turów (587.9–664.6 ppm) and FA from the electrofilter from Bełchatów (590.2–593.3 ppm). The largest share in REY was shown by LREY, whose content ranged from 77.3 to 85.0% of the total REY. The smallest share was by HREY, (1.9–3.2%) of the total REY (Table 7, Figure 3a,c). However, the REY enrichment efficiency after 4% HF treatment usually ranged from 29.73 to 71.30%, with the highest enrichment observed in the ashes from Turów (59.93–71.30%), and the lowest with the greatest variability in FA from the electrofilter from Bełchatów (7.60–38.55%). From these results, it can be concluded that the amount of dissolved REY in the residue after 4% HF ash treatment is lower than in the ashes from US power plants [32]. The BF2 sample with the lowest REY enrichment among the ashes from Bełchatów was significant. It contained the largest amounts of glass remaining after dissolution in 4% HF, which were difficult to interpret (Table 6, Figure 9).

A difference was observed between the SiO_2_ removal efficiency and the REY enrichment efficiency of the residue. This may be due to SiO_2_, Al_2_O_3_, and Fe_2_O_3_ being present in aluminosilicates and glass that were partially removed from the ashes (Figure 8). The study showed that the REY were present in the ash dissolution residue samples in similar proportions as in the raw ashes (Figure 3b,c).

The normalization curves for the ash dissolution residue samples from lignite combustion had similar shapes and characteristics (above the reference level) to the curves for raw ash (type L and L-M). The content of some REY in the samples was up to five times higher than in the UCC (Figure 6 and Figure 7). All the ash residue normalization curves from B showed positive anomalies for Eu and Y, in which Eu from Turów was larger than in the raw ash samples, while the anomalies for Y in the Turów samples were negative and smaller than for the raw ash (Figure 6 and Figure 7).

All ash dissolution residues in 4% HF were enriched in critical elements. Their content ranged from 99.6 to 201.5 ppm and for the ash from B, they consisted of 32.0–34.0% of the total REY, while the ash from Turów did not exceed 29% of the total REY.

The enrichment efficiency of critical elements in the residue samples was variable and ranged from 7.7 to 73.2%, with the highest being found in the samples from Turów (60.5–73.2%), while the samples from Bełchatów were lower (BA—29.6–50.3%; FA from the gas duct—34.6–37.9%), and the largest variations in enrichment efficiency (7.7–39.9%) were found for FA from the electrofilter. A much higher critical element enrichment efficiency of 60.5% was found for BA and 73.20% for FA in the Turów residue samples. All residue samples tested were also enriched in uncritical and excessive elements (Figure 4c). The study showed that critical, uncritical, and excessive elements were present in the residue samples after ash dissolution in 4% HF in similar proportions to the raw sample (Figure 4b,d).

The value of the prospective coefficient C_outl_ for the examined residue samples ranges from 0.70 to 0.92 for the ash from Bełchatów and 0.70–0.72 for the ash from Turów. The value of C_outl_ for almost all residue samples was the same or slightly higher than for the raw samples. All residue samples from Bełchatów are prospective REY raw materials, while the samples from Turów cannot be considered as such despite the highest enrichment efficiency (Table 7; Figure 5b).

### 3.6. REY and Its Relation to Main Chemical Components in Raw Ash Samples

**Figure 10:**
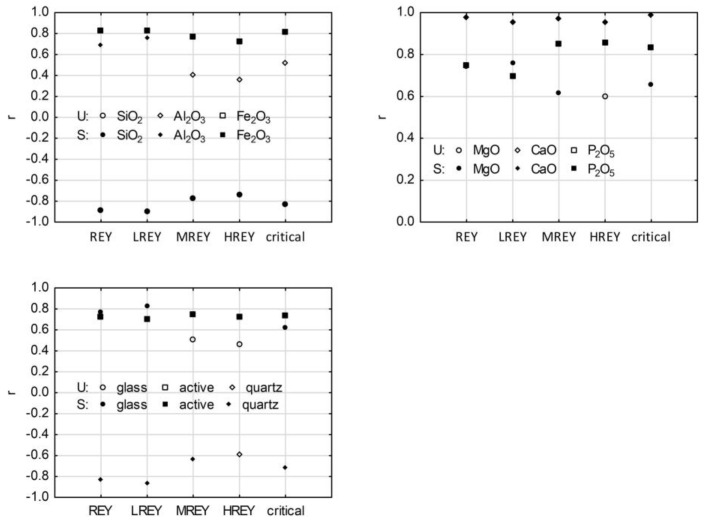
Values of correlation coefficients for relationships between the contents of REY, LREY, MREY, HREY, and critical elements and the contents of some chemical components and phases in the tested raw ash. Explanations: U—unsignificant correlation, S—significant correlation.

Significant positive correlations were observed between REY, LREY, MREY, and HREY contents and CaO, Fe_2_O_3_, and P_2_O_5_, amounting to r = 0.95–0.98, r = 0.73–0.83, and r = 0.70–0.86, respectively. The values of the correlation coefficients for the relationship with Fe_2_O_3_ decreased in the direction from LREY to HREY, and for the relationship with P_2_O_5_, they increased in the direction from LREY to HREY (Figure 10). Similarly, the content of CaO, Fe_2_O_3_, and P_2_O_5_ in ash samples showed a correlation with critical elements for which the values were r = 0.99, r = 0.82, and 0.84, respectively. There was a strong positive correlation between REY and MgO (r = 0.76), and the values of the correlation coefficients decreased from LREY to HREY (r = 0.76–0.60), but the correlations were significant only for LREY and MREY. An average significant positive correlation (r = 0.66) was found between the critical element and MgO, as well as for REY and Al_2_O_3_ (r = 0.69), and the values of the coefficients decreased from LREY to HREY (r = 0.76–0.36) but were significant only for the relationship with LREY. A strong negative correlation between REY and SiO_2_ (r = −0.88) was also observed, and its absolute coefficient values decreased from LREY to HREY (r = −0.90–0.73) and were significant, similar to those for the critical element and SiO_2_ (r = −0.82) (Figure 10).

### 3.7. REY and Its Relation to Phase Composition in Raw Ash Samples

A strong negative correlation between REY, LREY, and MREY and quartz was observed (r = −0.82, r = −0.86, and r = −0.63, respectively), but there was no significant correlation between HREY and quartz. The absolute values of the correlation coefficients for this relationship decreased from LREY to HREY. An average significant negative correlation was also found between the critical element and the quartz (r = −0.71) (Figure 10).

A strong positive correlation was observed between REY and LREY with amorphous glass (r = 0.77 and r = 0.83, respectively), but there was no significant correlation between MREY and HREY with glass. The coefficient values decreased from LREY to HREY. Similarly, significant positive correlations were found between active components and REY, LREY, MREY, HREY, and critical elements (r = 0.73, r = 0.70, r = 0.74, r = 0.73, and r = 0.74, respectively) (Figure 10). The same observations for quartz and glass were reported in US ashes [32,60,61,62].

### 3.8. REY and Main Chemical Components in Residue after Ash Dissolution in 4% HF

**Figure 11:**
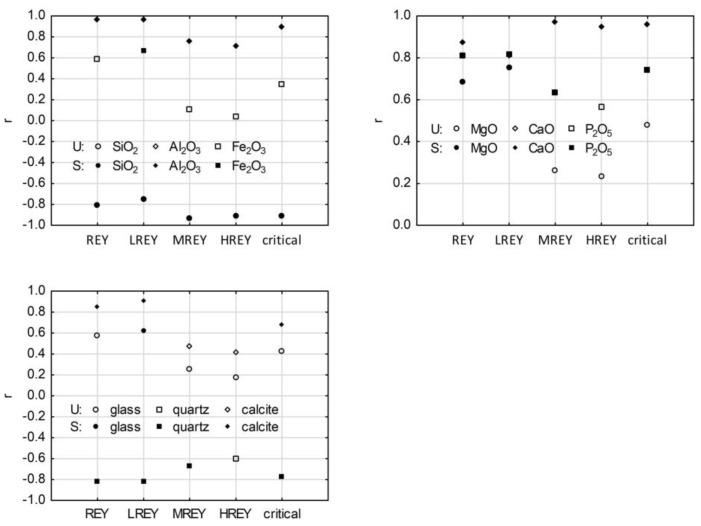
Values of correlation coefficients REY, LREY, MREY, HREY, and critical elements with some chemical components with phases in the residue samples after dissolution in 4% HF. Explanations: U—unsignificant correlation, S—significant correlation.

Correlations between REY, LREY, MREY, HREY, and critical element content and the main chemical constituents of ash residue samples after dissolution in 4%HF were analyzed. The results showed strong significant positive correlations of REY with CaO and P_2_O_5_ of r = 0.87 and r = 0.81, respectively, which increased from LREY to HREY (r = 0.81–0.95) and were all significant. In turn, for the relationship with P_2_O_5_, these values decreased from LREY to HREY (r = 0.82–0.57), but the correlations were significant only for LREY and MREY. The CaO and P_2_O_5_ also showed a correlation with critical elements, and the values of the correlation coefficients were r = 0.96 and 0.75, respectively (Figure 11).

Strong significant correlations were also found for REY, LREY, MREY, HREY, and critical elements with Al_2_O_3_ (r = 0.71–0.97), and the values of the correlation coefficients for REY decreased from LREY to HREY. A similar correlation between REY and Al_2_O_3_ was shown for ash from US power plants [61]. In contrast to the raw sample, a significant average positive correlation was found for Fe_2_O_3_ and LREY (r = 0.67). Significant positive correlations were found for REY and LREY with MgO, r = 0.68 and r = 0.75, respectively, while there were no significant correlations between MgO and the contents of HREY, MREY, and critical elements (Figure 11). The analyses showed that, as with the raw ash samples, there were strong negative correlations between REY content and SiO_2_ (r = −0.81). Similarly, significant strong correlations were found between LREY, HREY, MREY, critical elements, and SiO_2_ content.

### 3.9. REY and Its Relation to Mineral Components in Residue after Ash Dissolution in 4% HF

The analysis of the correlations coefficient values in the ash residue samples after 4% HF treatment was very similar to the raw samples: (i) a strong negative and significant correlation for REY, LREY, MREY, and critical elements with quartz (r = −0.82, r = −0.82, r = −0.67, and r = −0.76, respectively), with no significant correlation of HREY with quartz; and (ii) an average positive correlation of LREY with glass (r = 0.62), with no significant correlation of REY, MREY, HREY, and critical element with glass. Additionally, a strong positive correlation was found for REY, LREY, and critical elements with calcite (r = 0.85, r = 0.91, and r = 0.68, respectively), and the absolute values of these correlation coefficients decreased from LREY to HREY (Figure 11). These correlations were not found in the raw ash.

## 4. Conclusions

The assessment of the potential of the tested energy ashes as a source of REY must consider the concentrations of these metals and their relationship to the chemical, phase composition, and fuel. The study showed that in Bełchatów and Turów power plants, different quality lignite was burned: (i) low-rank B metalignite medium- or low-grade coal, and (ii) low-rank A subbituminous low-grade coal. The qualitative chemical and phase composition of the ashes was similar but quantitatively different. The ashes were classified based on the place of collection as follows: (i) BA was calsialic–medium acid and mixed–low pozzolanic (Bełchatów) and pozzolanic–medium pozzolanic, (Turów), (ii) FA from the electrofilter was calsialic–low acid and active–low pozzolanic (Bełchatów) and active–medium pozzolanic (Turów), and (iii) FA from the gas duct was calsialic–medium acid and inert–low pozzolanic.

- The REY distribution showed that FA from the electrofilters of Bełchatów contained the highest amounts of these elements (428–548 ppm), and the lowest were found in BA (228–247 ppm). The REY in FA from the flue gas duct was in amounts between the BA and the FA from the electrofilter (245–310 ppm). Ash from Turów did not show any differentiation because it occurred in amounts of 367 and 388 ppm (BA and FA, respectively).
- In the residue, after ash treatment in 4% HF, a high removal efficiency of almost all chemical components was usually observed compared to the raw samples. The exception was CaO because it enriched all ashes. The best presented high removal efficiency was for the main components: (i) SiO_2_–FA from electrofilters (ca. 50%), (ii) Al_2_O_3_–BA and FA from the gas channel (ca. 30%), (iii) Fe_2_O_3_–all ashes from power plant Bełchatów (ca. 40%), as well as glass from ashes from power plant Bełchatów (usually < 10%).

All residues after ash dissolution in 4% HF were enriched in REY, in the same proportions as in the raw ashes. The REY enrichment efficiency was varied, with the highest in the ash from power plant Turów (60–70%) and the lowest in the FA from the electrofilter of power plant Bełchatów (7–38%). The ash from power plant Bełchatów and its residue after dissolving in 4% HF were classified as promising REY raw materials, and they constitute the largest part of the total mass of ash generated in the power plant. The ash from power plant Turów was not considered a potential REY raw material despite the highest enrichment efficiency in the residues after dissolving in 4% HF.

The relationships of REY with the phases and chemical components revealed the possible recovery of these metals after the separation of active components and partially glass, as well as grains rich in CaO, P_2_O_5_, and Al_2_O_3_ in both raw ash samples and residue after dissolution in 4% HF for the extraction of REY, in particular, LREY.

## Figures and Tables

**Table 1:** The tested fuel and ash samples.

Power Plant	Sample Type (Waste Type)	Sample Symbol
Bełchatów	Lignite for power units 9–12	BW1
	Lignite for power units 5–8	BW2
	Lignite for power units 1–4	BW3
	Bottom ash—power unit 12	B1
	Bottom ash—power unit 8	B2
	Bottom ash—power unit 4	B3
	Fly ash gas duct—power unit 12	BL1
	Fly ash gas duct—power unit 8	BL2
	Fly ash—gas duct—power unit 4	BL3
	Fly ash—electrofilter—power unit 12	BF1
	Fly ash—electrofilter—power unit 8	BF2
	Fly ash—electrofilter—power unit 4	BF3
Turów	Lignite	TW4
	Bottom ash	T3
	Fly ash—electrofilter	T2

**Table 2:** Results of proximate and petrographic analysis of coal samples.

Sample	M^a^	A^d^	Q_s_^af^	H	L	I	SM	H^mmf^	L^mmf^	I^mmf^
	(%)	(%)	(MJ/kg)	(%)						
BW1	14.95	16.86	20.0	69	5	4	22	89	6	5
BW2	14.91	18.52	20.0	71	5	6	19	87	6	7
BW3	15.32	22.32	19.4	69	1	6	23	90	2	8
TW4	8.56	22.94	23.5	56	6	5	33	84	9	7

Explanation: Q_s_^af^—calorific value, M^a^ –moisture content, A^d^—ash content, H—huminite, L—liptinite, I—inertinite, mmf—mineral matter free state.

**Table 3:** The main chemical components’ content in the tested ash samples (in wt%).

Sample	SiO_2_	Al_2_O_3_	Fe_2_O_3_	MnO	MgO	CaO	Na_2_O	K_2_O	TiO_2_	P_2_O_5_	LOI	DAI	$\frac{SiO_{2}}{{Al}_{2}O_{3}}$	$\frac{\left(MgO+CaO\right)}{\left(K_{2}O+{Na}_{2}O\right)}$	$\frac{CaO}{MgO}$	$\frac{K_{2}O}{{Na}_{2}O}$
B1	52.49	15.57	5.03	0.03	0.68	11.47	0.08	0.09	1.04	0.18	13.34	3.91	3.37	70.35	16.85	1.13
B2	48.81	16.13	4.83	0.03	0.61	10.02	0.07	0.10	1.07	0.18	18.15	3.79	3.03	61.76	16.50	1.43
B3	49.42	15.17	5.12	0.03	0.59	11.17	0.07	0.09	0.92	0.22	17.19	3.71	3.26	72.44	18.98	1.29
BL1	54.00	17.84	4.15	0.03	0.62	11.21	0.08	0.10	1.05	0.27	10.64	3.95	3.03	64.72	18.10	1.25
BL2	58.49	16.88	4.52	0.03	0.55	9.62	0.07	0.10	0.96	0.24	8.56	4.63	3.47	60.29	17.64	1.43
BL3	47.13	19.90	6.71	0.04	0.64	13.57	0.07	0.11	1.04	0.34	10.47	3.06	2.37	77.72	21.21	1.57
BF1	44.17	20.81	5.84	0.05	1.10	20.72	0.13	0.13	1.27	0.24	5.54	2.20	2.12	82.69	18.91	1.00
BF2	36.12	22.82	7.46	0.07	1.09	27.39	0.16	0.10	1.02	0.56	3.21	1.45	1.58	107.73	25.18	0.63
BF3	35.10	22.72	7.27	0.07	1.07	26.94	0.11	0.11	1.07	0.50	5.02	1.49	1.54	125.00	25.19	1.00
T3	44.06	27.09	6.33	0.10	0.71	14.51	0.54	1.58	1.94	0.16	2.95	3.45	1.63	7.20	20.49	2.93
T2	39.21	27.64	5.55	0.05	1.49	17.43	1.29	1.34	2.22	0.20	3.60	2.42	1.42	7.21	11.68	1.04

**Table 4:** The phase components’ content in the tested ash samples (in wt%).

Sample	UC	G	Q	Mu	Ge	Cc	Ah	An	Mgh	He	Li
B1	13.34	31.8	27.8	5.5		6.0		13.7	1.9		
B2	18.15	34.0	23.8	6.0		4.6		12.7	0.8		
B3	17.19	30.1	26.8	6.7		5.3		11.8	2		
BL1	10.64	31.4	32.7	20.0	2.3	3.1					
BL2	8.56	35.4	37.3	14.0	1.9	2.9					
BL3	10.47	47.5	22.7	12.0	3.5	3.9					
BF1	5.54	51.7	15.9		7.5	3.3	5.1	11.1			
BF2	3.21	59.5	8.6		12.1	3.1	3.2	10.4			
BF3	5.02	58.3	8.7		10.2	4.1	4.9	8.9			
T3	2.95	70.7	10.3			5.4	10.6				
T2	3.60	66.3	6.3			5.8	12.4			3.08	2.45

Explanations: UC—unburned carbon, G—glass, Q—quartz, Mu—mullite, Ge—gehlenite, Cc—calcite, Ah—anhydrite, An—anorthite, Mgh—maghemite, He—hematite, Li—lime.

**Table 5:** The REY content in examined ash samples.

Sample	Y	La	Ce	Pr	Nd	Sm	Eu	Gd	Tb	Dy	Ho	Er	Tm	Yb	Lu
	(ppm)														
B1	34	49.2	89.3	9.95	37.6	7	1.43	5.4	0.8	5.1	1	3	0.42	2.9	0.45
B2	28	46.5	84.7	9.36	35.1	6.5	1.28	4.9	0.8	4.6	0.9	2.7	0.4	2.7	0.41
B3	30	48.1	88.4	9.77	37.3	6.8	1.33	5.1	0.8	4.7	0.9	2.7	0.41	2.7	0.41
BL1	36	54.3	101	11.2	42.5	8	1.56	6.1	1	5.7	1.1	3.3	0.48	3.2	0.52
BL2	30	48.6	90.4	10.1	38.8	7.2	1.36	5.2	0.8	4.9	1	3	0.44	2.9	0.44
BL3	40	60.5	115	12.6	48	9.1	1.8	6.9	1.1	6.5	1.2	3.5	0.52	3.3	0.55
BF1	60	84.2	151	17	64.7	12.6	2.6	10.9	1.7	9.5	1.9	5.6	0.79	4.9	0.81
BF2	73	103	196	22.1	88.6	17.1	3.49	13.7	2.1	12.2	2.4	6.8	0.94	6.1	0.95
BF3	68	95.5	174	19.4	76.9	15	2.93	13	1.9	11.1	2.1	6.1	0.82	5.5	0.84
T3	29	85.1	141	16.4	59.2	11.4	2.34	8.1	1.2	6.5	1.1	3	0.41	2.5	0.36
T2	34	85.7	150	16.4	59.9	11.4	2.54	9.5	1.5	7.9	1.4	3.6	0.48	3.2	0.46
**Sample**	**REY**	**LREY**	**MREY**	**HREY**	**LREY**	**MREY**	**HREY**	**Critical**	**Uncritical**	**Excessive**	**Critical**	**Uncritical**	**Excessive**	**C_outl_**	
	**(ppm)**				**(%)**			**(ppm)**			**(%)**				
B1	247.6	193.1	46.7	7.8	78.0	18.9	3.1	81.9	71.6	94.1	33.1	28.9	38.0	0.87	
B2	228.9	182.2	39.6	7.1	79.6	17.3	3.1	72.5	67.3	89.1	31.7	29.4	38.9	0.81	
B3	239.4	190.4	41.9	7.1	79.5	17.5	3.0	76.8	69.8	92.8	32.1	29.1	38.8	0.83	
BL1	276.0	217.0	50.4	8.6	78.6	18.2	3.1	90.1	79.6	106.3	32.6	28.8	38.5	0.85	
BL2	245.1	195.1	42.3	7.8	79.6	17.2	3.2	78.9	71.1	95.2	32.2	29.0	38.8	0.83	
BL3	310.6	245.2	56.3	9.1	79.0	18.1	2.9	100.9	89.1	120.6	32.5	28.7	38.8	0.84	
BF1	428.2	329.5	84.7	14.0	77.0	19.8	3.3	144.1	124.7	159.4	33.7	29.1	37.2	0.90	
BF2	548.5	426.8	104.5	17.2	77.8	19.1	3.1	186.2	155.9	206.4	33.9	28.4	37.6	0.90	
BF3	493.1	380.8	96.9	15.4	77.2	19.7	3.1	166.9	142.9	183.3	33.9	29.0	37.2	0.91	
T3	367.6	313.1	47.1	7.4	85.2	12.8	2.0	101.2	121.0	145.4	27.5	32.9	39.5	0.70	
T2	388.0	323.4	55.4	9.1	83.4	14.3	2.4	109.4	123.0	155.5	28.2	31.7	40.1	0.70	

**Table 6:** Dissolution efficiencies of tested ash samples after 4% HF treatment.

Sample	SiO_2_	REY	Glass	H-SiO_2_	H-REY	H-Glass	R-SiO_2_	E-REY	R-Glass
	(%)	(ppm)	(%)	(%)	(ppm)	(%)	(%)	(%)	(%)
B1	52.49	247.6	31.8	40.55	364.8	6.52	22.74	47.34	79.48
B2	48.81	228.9	34.0	38.82	312.5	7.29	20.46	36.57	78.52
B3	49.42	239.4	30.1	38.78	310.6	9.18	21.53	29.73	69.49
BL1	54	276	31.4	41.75	375.1	8.77	22.68	35.94	72.03
BL2	58.49	245.1	35.4	47.04	336.6	5.07	19.57	37.33	85.70
BL3	47.13	310.6	47.5	32.79	428.2	5.98	30.43	37.87	87.41
BF1	44.17	428.2	51.7	22.62	593.3	5.68	48.78	38.55	89.01
BF2	36.12	548.5	59.5	18.41	590.2	20.45	49.01	7.6	65.62
BF3	35.1	493.1	58.3	14.07	592.9	9.04	59.9	20.25	84.49
T3	44.06	367.6	70.7	36.37	587.9	24.73	17.46	59.93	65.04
T2	39.21	388	66.3	25.16	664.6	14.61	35.83	71.3	77.96

Explanations: E—enrichment; R—reduction; H—samples after 4% HF treatment.

**Table 7:** The REY content in the residue samples after dissolution of the ashes in 4% HF.

Sample	Y	La	Ce	Pr	Nd	Sm	Eu	Gd	Tb	Dy	Ho	Er	Tm	Yb	Lu
	(ppm)														
B1	52	70.8	130	15.1	56	10.3	2.04	8.2	1.3	7.5	1.5	4.3	0.63	4.4	0.68
B2	40	63.1	115	13	47.6	8.7	1.76	7	1	6.1	1.2	3.5	0.52	3.5	0.56
B3	40	61.8	115	12.7	47.2	8.7	1.78	7.3	1	6.2	1.2	3.4	0.49	3.3	0.54
BL1	49	74	138	15.5	56.9	10.6	2.24	8.9	1.3	7.6	1.5	4.2	0.61	4.1	0.68
BL2	43	66.2	124	14.1	51.7	9.6	1.98	7.9	1.2	6.8	1.4	3.9	0.56	3.7	0.6
BL3	56	83.8	158	17.6	66	12.2	2.53	10.2	1.5	8.4	1.6	4.7	0.65	4.3	0.7
BF1	83	115	209	24.7	92.8	17.7	3.63	14.7	2.2	12.8	2.4	7.1	0.97	6.3	0.95
BF2	82	111	211	24.1	93.4	17.9	3.69	15.6	2.2	12.5	2.4	6.7	0.93	5.8	0.94
BF3	84	114	210	24.3	92	17.8	3.67	15.6	2.2	12.5	2.4	6.7	0.92	5.9	0.94
T3	48	135	226	26.9	94	17.7	3.64	13	2	10.3	1.7	4.6	0.61	3.9	0.58
T2	59	148	254	29.4	106	20.1	4.27	15.1	2.2	12.5	2.1	5.6	0.73	4.9	0.7
**Sample**	**REY**	**LREY**	**MREY**	**HREY**	**LREY**	**MREY**	**HREY**	**Critical**	**Uncritical**	**Excessive**	**Critical**	**Uncritical**	**Excessive**	**C_outl_**	
	**(ppm)**				**(%)**			**(ppm)**			**(%)**				
B1	364.8	282.2	71.0	11.5	77.4	19.5	3.2	123.1	104.4	137.2	33.8	28.6	37.6	0.90	
B2	312.5	247.4	55.9	9.3	79.2	17.9	3.0	100.0	91.8	120.8	32.0	29.4	38.6	0.83	
B3	310.6	245.4	56.3	8.9	79.0	18.1	2.9	99.6	90.5	120.5	32.1	29.1	38.8	0.83	
BL1	375.1	295.0	69.0	11.1	78.6	18.4	3.0	121.2	109.0	144.9	32.3	29.1	38.6	0.84	
BL2	336.6	265.6	60.9	10.2	78.9	18.1	3.0	108.6	97.8	130.3	32.3	29.1	38.7	0.83	
BL3	428.2	337.6	78.6	12.0	78.8	18.4	2.8	139.1	123.8	165.3	32.5	28.9	38.6	0.84	
BF1	593.3	459.2	116.3	17.7	77.4	19.6	3.0	201.5	172.1	219.6	34.0	29.0	37.0	0.92	
BF2	590.2	457.4	116.0	16.8	77.5	19.7	2.8	200.5	168.6	221.1	34.0	28.6	37.5	0.91	
BF3	592.9	458.1	118.0	16.9	77.3	19.9	2.8	201.1	171.7	220.2	33.9	29.0	37.1	0.91	
T3	587.9	499.6	76.9	11.4	85.0	13.1	1.9	162.5	192.6	232.8	27.6	32.8	39.6	0.70	
T2	664.6	557.5	93.1	14.0	83.9	14.0	2.1	189.6	212.6	262.4	28.5	32.0	39.5	0.72	

## Data Availability

The original contributions presented in the study are included in the article, further inquiries can be directed to the corresponding author.
